# When Density Matters: Hydraulic and Salinity Evolution in Groundwater‐Fed Pit Lakes in Semiarid and Arid Climates

**DOI:** 10.1111/gwat.70074

**Published:** 2026-05-07

**Authors:** Birte Moser, Peter G. Cook, Janek Greskowiak, Ilka Wallis

**Affiliations:** ^1^ Flinders University, College of Science and Engineering, National Centre for Groundwater Research and Training P.O. Box 2100 Adelaide South Australia 5001 Australia; ^2^ Department of Biology and Environmental Sciences Carl‐von‐Ossietzky University of Oldenburg Ammerländer Heerstraße 11 Oldenburg D‐26129 Germany

## Abstract

Although groundwater flow directions are influenced by hydraulic gradients and density gradients, density gradients are often assumed negligible outside coastal zones and saline lake environments. Density gradients are rarely considered in mining studies, but can alter groundwater flow patterns, and cause outflow of water from pit lakes despite inward hydraulic gradients. Open mine pits often intersect regional water tables, requiring dewatering during mining operations. At the end of mine life, groundwater abstraction ceases, frequently leading to the development of pit lakes. Due to prolonged water residence time, and evaporation, pit lake water quality may deteriorate with salinization being a common problem. While salinity differences between pit lake water and groundwater are small initially, they increase with time, inducing density contrasts. Consequently, dense pit lake water may move along density gradient towards less dense groundwater. This changes the flow patterns around the pit lake and impact on surrounding aquifer water quality. In this study, we advance process understanding of how density effects alter flow and salinity patterns in pit lake environments post‐mining using numerical modeling. We show the impact of ambient groundwater salinity, regional hydraulic gradients, evaporation rates, and hydraulic conductivities on the interaction between a pit lake and the surrounding aquifer. We demonstrate how density effects can substantially increase lake water outflow and decrease pit lake water salinities. Pit lakes can turn from terminal sinks into throughflow systems purely due to variable‐density flow. Understanding the hydraulic and salinity evolution of pit lakes is crucial for planning post mining rehabilitation.

## Graphical abstract

Pit lakes in semiarid and arid regions are often assumed to be terminal sinks for groundwater following mine closure. Using synthetic numerical modeling, we demonstrate that evaporation‐driven density gradients can reverse this behavior, transforming pit lakes into throughflow systems and potentially increasing salinity in adjacent aquifers. Findings can improve mine closure practice.

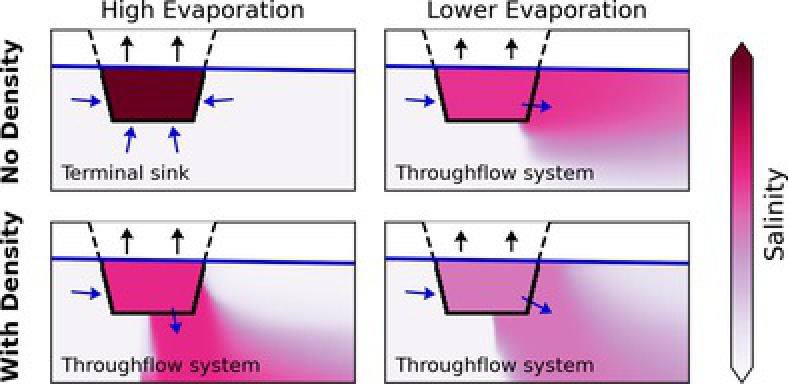

## Introduction

Groundwater flow directions are influenced by both hydraulic gradients and water density gradients (Post and Simmons 2022). Often, density gradients are assumed to be negligible outside coastal zones (Simmons 2005; Doulabian et al. 2024), sedimentary basins (Alkalali and Rostron 2003) and saline lake environments (Simmons and Narayan 1997), and groundwater flow modeling is based only on hydraulic considerations. However, the range of environments where density contrasts can alter flow patterns may be greater than traditionally considered. One such environment is open pit mining areas, where the mined ore is accessed from the surface of the land via the construction of a pit that extends below the regional water table, and mine dewatering is required. Open pit mines account for large areas of mining land use and are common around the globe (Werner et al. 2020; Liang et al. 2021; Maus and Werner 2024). Following mining, dewatering is no longer required, and the abstraction of groundwater ceases. This frequently has led to the development of pit lakes, either historically, when mine closure was not managed and mines abandoned (Kumar et al. 2009; Soni et al. 2014; Venkateswarlu et al. 2016; de Lange et al. 2018), or intentionally as the preferred mine closure option. In Australia, for example, about 350 open pit mines are currently active (Australian Government—Geoscience Australia 2024) and as Australia transitions from a mining boom in the early 2000s, it is expected that about 160 open mines are due to close by 2040, with a vast number of these predicted to develop into pit lakes (CSIRO 2023).

In semiarid and arid climates, pit lakes are typically groundwater‐fed (Newman et al. 2020; Johnstone 2021). Because of high evaporation rates, which can reach up to 4 m/year in these climates, there is a common belief that these pit lakes remain terminal sinks, that is, groundwater enters the pit, but water loss only occurs due to evaporation (e.g., Zhao et al. 2010). This is significant, because in this terminal sink scenario, downgradient aquifers would be unaffected by pit lake water discharge, and this assumption influences the closure options mines pursue. Dissolved salts and trace metals may enter pit lakes through groundwater inflow, leaching, or direct interaction with pit walls, thereby degrading water quality. Under terminal sink conditions, prolonged water residence times combined with evaporation and evapoconcentration further exacerbate this deterioration (Castendyk et al. 2015; Roemer et al. 2015; Lund and Blanchette 2021).

For example, monitoring data from the Mount Goldsworthy pit lake, Western Australia, a former iron ore mine, show an increase in salinity from 1400 to 5500 mg/L total dissolved solids (TDS) over a 14‐year period (Johnson and Wright 2003). In the Berkeley Pit, Butte, Montana, 7–17 years after flooding began, the mean specific conductance of the pit lake water was 7340 μS/cm, compared to 680 to 1650 μS/cm in the surrounding groundwater (averaged values) (Pellicori et al. 2005).

Lakes that intersect with the regional water table can be terminal groundwater sinks. However, salinity will increase over time, increasing the likelihood of outflow from pit lakes. Lake outflow will occur if density gradients overcome hydraulic gradients. While pit lakes are typically terminal sinks for the surrounding groundwater system immediately after mining and dewatering ceases, throughflow systems can form as the groundwater recovers and a background hydraulic gradient re‐establishes (Moser et al. 2024). Under throughflow conditions, groundwater enters the pit lake while pit lake water exits the mine void downgradient and can impact the water quality of the receiving aquifer (Savage et al. 2000; Kuznetsova and Ivanov 2019; Jost et al. 2024). Under low hydraulic conductivities of the surrounding geology, low hydraulic gradients and high evaporation rates, if density is not considered, pit lakes tend to remain terminal sinks (Moser et al. 2024). But these systems may actually transition into throughflow systems when density‐driven leakage is considered (Simmons and Narayan 1997; McCullough 2024): an increase in salinity, and with that an increase in density, could cause pit lake water to outflow along the negative density gradient into the underlying aquifer.

Detecting outflowing pit lake water through monitoring wells suffices to classify a pit lake as a throughflow system. Conversely, not detecting outflowing pit lake water is not sufficient to classify a pit lake as a terminal sink: It is impossible to sample the whole aquifer surrounding the pit, and therefore outflow could go undetected. Consequently, throughflow systems can be classified as such through either field measurement or conceptualization (modeling), while classifying pit lakes as terminal sinks would need to rely upon conceptualizations or modeling studies.

Lund et al. (2012) investigated the hydrogeological regime of pit lake galleries in the Collie pit lake district, Western Australia, and observed those to be generally throughflow systems, with the exception of very dry seasons when pit lakes transformed into terminal sinks. Lake Kepwari in Western Australia was also found to act as a throughflow system through modeling (Salmon et al. 2017). In some pit lake environments, the adjacent groundwater was either affected or potentially affected due to outflowing water from the pit (Savage et al. 2000; Kuznetsova and Ivanov 2019).

Examples of mines that are modeled to become terminal sinks are the Nifty Copper Operation, Aditya Birla and Tallering Peak Iron Ore Mine, Mount Gibson Mining the Kışladağ Gold Mine, Uşak in Turkey and the Kintyre Uranium open‐pit in Western Australia and in the Waterberg Coal‐field (McCullough et al. 2013; Roemer et al. 2015; Unsal and Yazicigil 2016). Density‐dependent flow and transport is frequently considered, for example in the context of shallow salt disposal evaporation ponds, submarine groundwater discharge or saltwater intrusion in coastal areas (Meisler et al. 1984; Simmons and Narayan 1997; Wooding et al. 1997; Greskowiak 2014; Hamann et al. 2015). Surprisingly, however, there are limited studies that consider the salinity development and resulting density effects of developing mine pit lakes in terms of enhancing seepage from pit lakes into groundwater (Roemer et al. 2015). Modeling studies that predict the evolution of post‐mining environments typically assume density‐independent flow even under predicted high rises in salinity (e.g., Peak Iron Mines 2021; JBS&G 2022). In many cases, lake salinity concentrations are estimated using water budgets rather than solute transport models, making the inclusion of density‐dependent flow a considerably more complex undertaking. This may partly be a consequence of the notable lack of publicly available data on long‐term monitoring records for pit lake water chemistry, due to confidentiality concerns. Thus, with a few exceptions (e.g., Johnson and Wright 2003; Gammons and Duaime 2006; Mollema et al. 2015; Salmon et al. 2017), our understanding of long‐term hydraulic behavior and water chemistry evolution in pit lakes and the interaction with adjacent aquifers post‐closure remains limited, hampering closure planning. Given the scarcity of publicly available long‐term monitoring data, numerical simulations play a crucial role in improving process understanding (Szczepiński 2019) and help bridge the knowledge gap where empirical data is lacking. Prominent past studies pursuing this approach have proven to be highly instructive by elucidating dominant processes, testing hypotheses, and guiding future data collection efforts (e.g., Stafford et al. 1998; Marshall et al. 2020).

This study aims to increase the basic process understanding of how density‐dependent flow can influence the evolution of salinity in deep lakes in semiarid and arid climates. We focus on open pit mining environments, where density‐dependent flow is rarely considered in mine closure planning. We are only aware of very few modeling studies that have considered density‐dependent flow in mine closure (Yager et al. 2009; Luo et al. 2011; Roemer et al. 2015), and there is still little general understanding of how water density influences groundwater flow processes in these environments. Here, we utilize synthetic numerical models to simulate real‐scale mining environments. The contamination of adjacent aquifers as well as the salinity concentrations in the pit lake are examined under consideration of ambient groundwater salinities, regional hydraulic gradients and hydraulic conductivities of diverse mine environments. The work provides guidance for practitioners and regulatory agencies as to when density‐driven flow should be considered.

## Methods

Numerical models were set up to determine the predominant environmental factors and processes that influence the evolution of salinity in groundwater‐fed pit lakes. The environmental conditions broadly reflect the semiarid to arid conditions of the Pilbara region in Western Australia, which are comparable to other large mining regions in parts of South Africa and the United States (Newman et al. 2020; Johnstone 2021). The Pilbara is an active mining area in Australia, particularly well known for large iron ore deposits and open pit operations that take place below the water table (Skrzypek et al. 2013).

**Figure 1:**
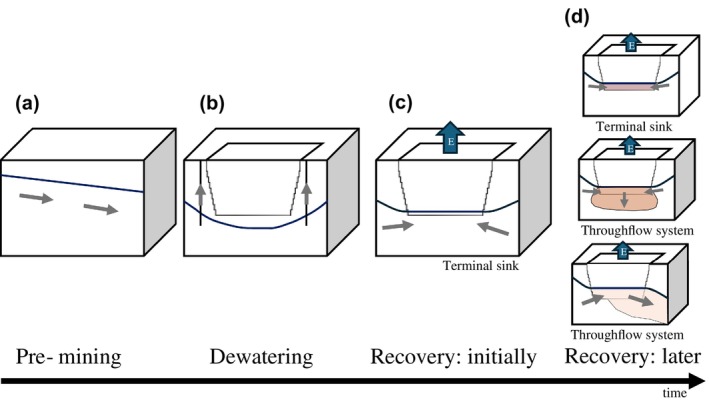
Schematic diagram of simulated mining phases: (a) Pre‐mining: hydrogeological system under steady‐state conditions prior to abstraction; (b) Dewatering: transient with pumping to dewater the pit; and (c, d) Recovery: post‐mining, abstraction ceases, and the water table recovers.

An evolving pit lake was simulated in three phases: pre‐mining, dewatering, and recovery (Figure 1). Flow simulations were set up for all three operational phases, however, during groundwater recovery, a solute transport model was included. To facilitate the investigation of density effects and ensuing flow patterns in the developing pit lake and surrounding aquifer, simulations with and without the consideration of density‐driven flow were compared.

### Numerical Model Approach

Numerical groundwater modeling was conducted using Modflow 6 (version 6.4.2) which utilizes a finite control volume method (Langevin et al. 2017). A groundwater flow model (GWF) was coupled with a groundwater transport model (GWT). Fluid density was implemented using the buoyancy package (BUY). The pit lake was modeled via the Lake package (LAK6) (Merritt and Konikow 2000), allowing the simulation of flow interaction between the lake and aquifer. Modflow 6 enables the lake package to be used in combination with the transport model and buoyancy package via the lake transport package (LKT) (Mancewicz et al. 2023). By implementing the LKT package, the exchange of solutes between the aquifer and lake needed to be simulated under the assumption of perfect mixing within the lake. Models were set up and results were post‐processed using FloPy version 3.4.2 (Bakker et al. 2024) and Matplotlib version 3.5.2 (Hunter 2007).

### Flow Model

**Figure 2:**
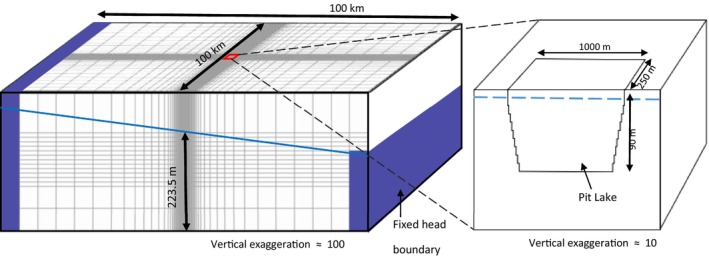
Scheme of the model setup. The water table (light blues) is shown for pre‐mining conditions. Note that the pit lake is only implemented into the model during the recovery phase, the water table is shown as dashed line for reference.

The pre‐mining phase was set up to simulate the established hydraulic background conditions prior to the commencement of mining. This phase was simulated in steady state and fixed head boundaries were implemented at the upgradient and downgradient domain boundaries to establish the hydraulic background gradient (Figure 2). The transient dewatering phase (20 years) simulated the duration of the mining operation. In this phase, 20 wells with abstraction rates of 6000 m^3^/day, each, dewatered the mine pit, emulating a typical dewatering operation in the Pilbara. The wells were equally distributed around the pit, 50 m from the mine pit rim. As the water table was assumed to always remain below the deepest mining bench, the mine void was not implemented as an explicit model feature during the dewatering phase of the numerical model. At the commencement of the recovery phase, the wells were switched off and the lake package was implemented in the location of the excavated mine pit, allowing a pit lake to emerge in accordance with the rebound of the groundwater levels. Evaporation was allowed to occur from the expanding pit lake surface. The recovery phase was simulated transiently for 500 years. To avoid interference with model boundaries the model domain was large in comparison to the pit dimensions, with the entire domain measuring 100 km x 100 km with a depth of 225 m. The lower domain boundary thereby represented the lower aquifer base, which resulted in an aquifer thickness of about 225 m at the pit lake, which was found to be a realistic value for the region. A rectangular pit of 1000 m × 250 m (at the water table) and a depth of 90 m was placed in the center of the model domain; thereby the pit depth always refers to the pit depth below water table. Pit walls sloped at an angle of 39° leading to a pit base area of 800 m × 25 m. The re‐wetting of model cells was facilitated through the Newton–Raphson formulation.

Model parameters for the flow simulation are listed in Table 1. Figure 1 presents a schematic of the modeled periods, while Figure 2 shows the general model setup, closely aligning with that in Moser et al. (2024).

**Table 1:** Model Parameters for the Flow and Transport Model Setup

Parameter	Unit	Value
Flow model		
Dx, Dy	m	12.5–5000
Model dimensions (length × width × depth)	km	100 × 100 × 0.225
Dz	m	10–90^a^
Minimum time step dt (adaptive time stepping)	d	10
Specific yield	‐	0.1
Specific storage	1/m	1 × 10^−4^
Effective porosity	‐	0.25
Salinity slope (BUY)^b^	kg/g	7 × 10^−4^
Density reference	kg/m^3^	1000
Transport model		
Longitudinal dispersivity	m	8
Transversal dispersivity	m	0.8
Molecular diffusion coefficient	m^2^/d	4.32 × 10^−5^
Advection scheme	‐	Upstream weighting
Base case		
Horizontal hydraulic conductivity K_h_	m/d	4
Vertical hydraulic conductivity K_v_	m/d	0.4
Pit geometry (length × width × depth)	m	1000 × 250 × 90
Pit wall slope	degrees	39
Net evaporation rate^c^	m/year	1
Pre‐mining hydraulic gradient	‐	1 × 10^−3^
Initial concentration in aquifer (TDS) C_0_	mg/L = g/m^3^	1550

Note: Dx, Dy, and Dz are the length, width, and depth of the model cells, respectively. Linear change in density, dependent on the salinity concentration C_0_, is calculated within the buoyancy package (BUY) using the salinity slope. The listed aquifer parameter values are given for the base case.

^a^ Note that the top layer has a thickness (Dz) of 90 m to allow for establishing the regional hydraulic gradient for various scenarios, while maintaining a saturated thickness of 223.5 m at the mine site under pre‐mining conditions (Figure 2).

^b^ Calculated as $\frac{\rho_{\text{saltwater}}-\rho_{\text{freshwater}}}{C_{\text{saltwater}}-C_{\text{freshwater}}}=\frac{1025\;\frac{\mathrm{kg}}{m^{3}}-1000\;\frac{\mathrm{kg}}{m^{3}}}{35,000\;\frac{g}{m^{3}}-0\;\frac{g}{m^{3}}}=0.0007\frac{\mathrm{kg}}{g}=0.7.$

^c^ The net evaporation term is evaporation minus precipitation minus runoff.

### Transport Model

The transport model was implemented during the recovery phase to simulate the change in salinity in the pit lake and the surrounding groundwater system due to evapoconcentration of pit lake water. The initial concentration of total dissolved solids (TDS) in the groundwater system was set to C_0_ = 1550 mg/L. During the recovery of the water table, as groundwater filled the pit, the pit lake surface area increased through time. Pit lake water increased in concentration through evapoconcentration. The upgradient model boundary conditions were constant concentration (C_0_).

To evaluate the impact of changes in density, model simulations were performed with and without the consideration of density‐dependence. The flow and transport models, including the lake and lake transport package, were coupled. This allowed the exchange of concentration between the lake and the groundwater system. Where variable density was considered, the buoyancy package was activated. Within the developing pit lake, perfect mixing of the lake water was assumed, a valid approach for pit lakes that undergo mixing at least once a year (holomictic conditions). These conditions are expected to occur frequently in semiarid and arid climates. This is discussed in detail in later sections (see Hydraulic Gradient).

### Model Scenarios

To determine the impact of density on pit lake–aquifer interaction, scenarios with varying environmental settings were investigated. Parameters were altered and changes in pit lake concentrations and patterns of pit lake outflow and salinity plumes were explored. This was done for model realizations with and without density consideration. The investigated parameters and their ranges are chosen to loosely capture conditions in the Pilbara:

- Hydraulic conductivity (K_h_) was varied between 0.1 and 4 m/d (Skrzypek et al. 2013) and the anisotropy factor (K_h_/K_v_) was set to 1 or 10.
- Net evaporation rate (E), which is evaporation minus precipitation minus runoff, was varied between 1 and 4 m/year (Sudmeyer 2016; Bureau of Meteorology 2024)
- Initial groundwater concentrations (C_0_) were between 250 and 7500 mg/L (Skrzypek et al. 2013; Cook et al. 2017)

Pit geometries are formed around the occurrence of the mined resource and are therefore highly variable (Werner et al. 2020; Maus et al. 2022). A pit size for a small to medium size pit of 1000 m × 250 m × 90 m was set up as base case (e.g., RPS Aquaterra 2013). The pit depth (d) was varied between 30 and 90 m depth. Additionally, a scenario with a 90°‐rotated pit (orthogonal to groundwater flow) as well as a scenario with a larger pit lake surface area (A_SF_) of 2 km × 2 km was set up. The regional hydraulic gradient, implemented through fixed head boundaries (Figure 2), was varied between 0 and 0.002. Although a zero gradient is a theoretical construct, gradients can be very low in quasi‐stagnant groundwater systems. The zero‐gradient scenario was included deliberately as it is a common assumption in conceptual models of mine sites and reflects an endmember for evaluating the influence of hydraulic gradient variations. Recharge, and seasonal fluctuations in net evaporation were neglected after model simulations confirmed that seasonal climate and pit lake water table fluctuations did not alter the long‐term pit lake salinity behavior (model simulations not shown).

## Results

### Effect of Density‐Dependent Flow on Pit Lake and Aquifer Salinity

**Figure 3:**
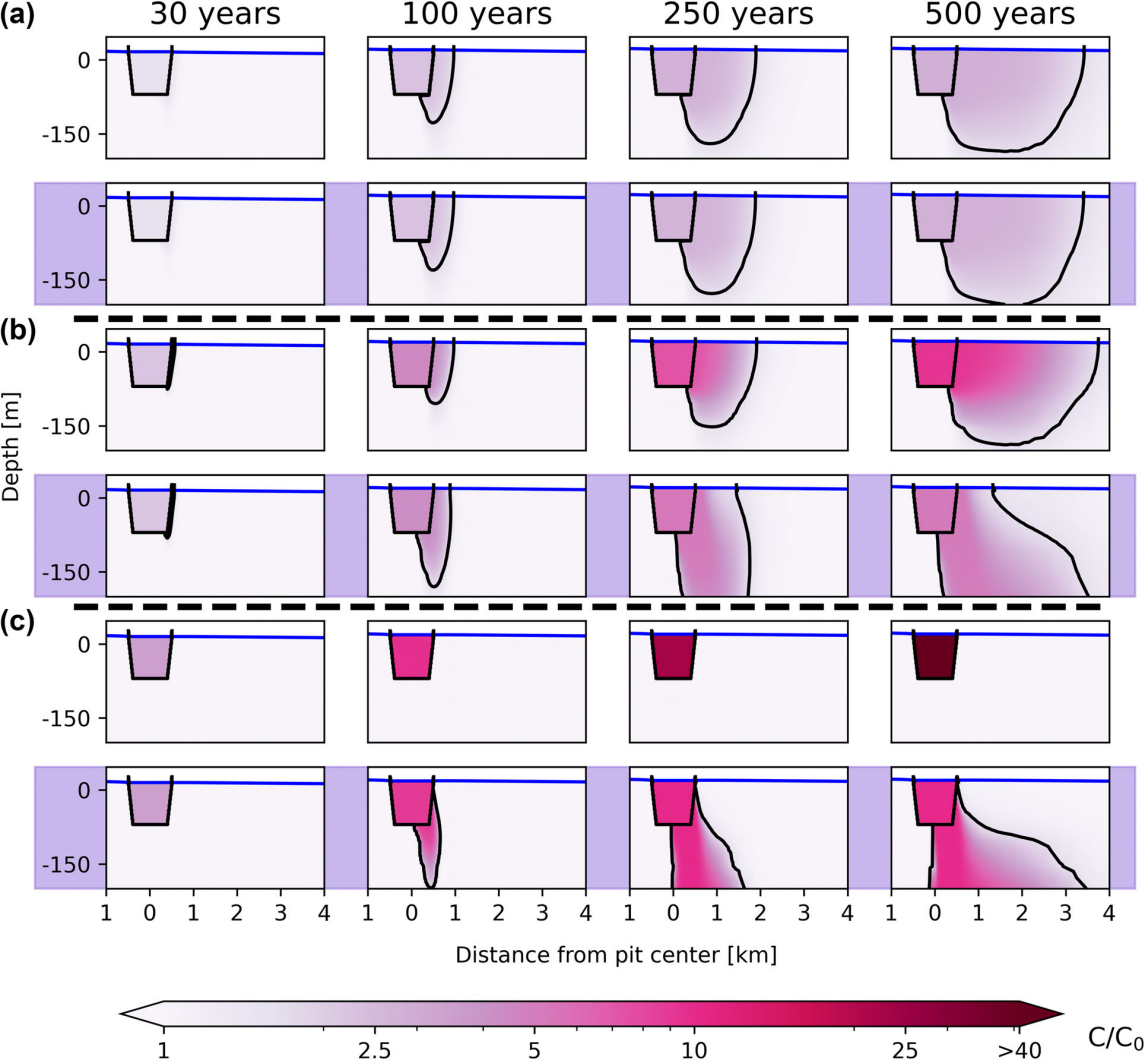
Temporal development of pit lake salinity under consideration of density (purple background) and under conservative transport assumption, that is, without density consideration (white background). The scenarios were selected to reflect the varying impact by density inclusion rather than a single‐parameter sensitivity. The selected scenarios are: (a) background groundwater concentration of 250 mg/L and net evaporation rate of 1 m/year; (b) background groundwater concentration of 1550 mg/L and net evaporation rate of 2 m/year; and (c) background groundwater concentration of 1550 mg/L and net evaporation rate of 4 m/year. All other parameters as per base case model setup (Table 1). For easier comparison, isolines are shown for C = 1.6C_0_ (black line).

Following mine closure, the salinity of a developing groundwater‐fed pit lake changes over time depending on the interplay between groundwater inflow and evaporation from the lake surface. Shortly after mining ceases, pit lake salinity is similar to the salinity of inflowing groundwater. With time, however, prolonged residence times of water in the pit lake and ongoing evapoconcentration from the increasing pit lake surface area, pit lake salinities increase. The evolution of pit lake and aquifer concentrations are shown for three different mine environments in Figure 3.

Under low groundwater background concentrations (C_0_ = 250 mg/L; Figure 3a) density effects remain small within the 500 years simulation time, and no significant differences between the realizations with and without density are observable. Both models rapidly become throughflow systems and the concentration of water within the pit lake increases about 2.5 times (i.e., to 700 mg/L) after 500 years. With time, the outflowing pit lake water affects an ever‐increasing volume of the groundwater system.

With increasing evaporation (Figure 3b), density‐dependent flow becomes increasingly important and affects both pit lake water salinity and affected aquifer volume. Pit lake salinities thus remain relatively lower when density is considered, as outflow from the pit lake is enhanced due to density driven flow, resulting in shorter water residence times within the lake.

Interestingly, at very high evaporation (4 m/year; Figure 3c), the pit lake becomes a terminal sink if density is not considered. As a consequence, pit lake salinities increase rapidly with time, while the aquifer remains unaffected. In these cases, however, consideration of density‐dependent flow changes the flow system entirely; outflow due to the increasing density gradient between pit lake and aquifer transforms the pit lake into a throughflow system. Pit lake salinities are lower, but the volume of aquifer impacted by saline pit lake water outflow is greater.

**Figure 4:**
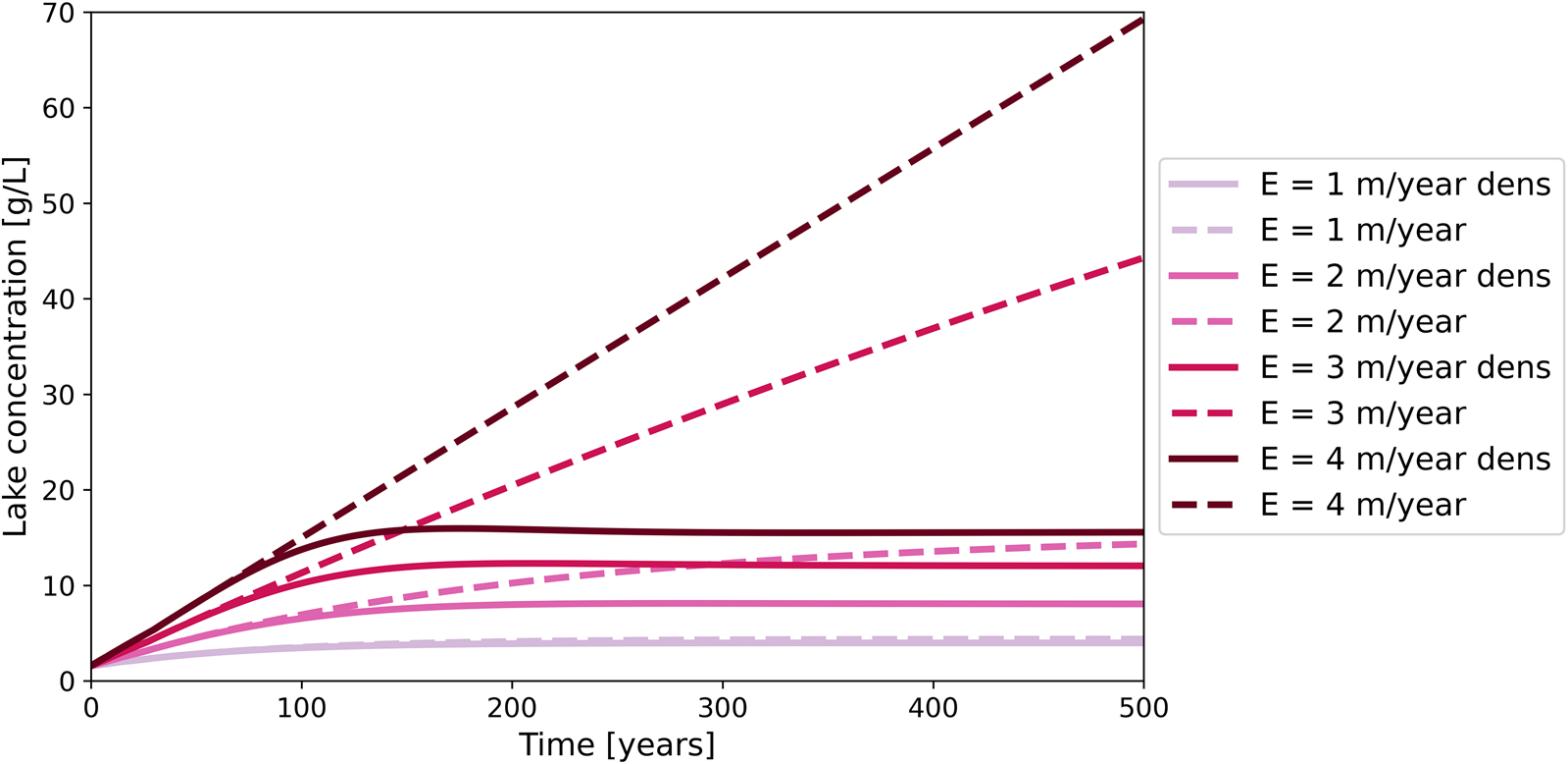
Temporal changes in pit lake concentrations after mining operations cease for different evaporation rates (E). Solid lines show scenarios where density‐dependent flow is included and dashed lines show scenarios without consideration of density effects. Initial groundwater concentrations C_0_ = 1550 mg/L = 1.55 g/L in all scenarios.

The impact on the aquifer by outflowing pit lake water generally increases when density‐dependent flow is considered in the numerical model. However, pit lake salinities increase less than predicted under density‐independent flow assumptions (Figure 4). For scenarios where density‐driven flow is not considered (dashed lines), concentrations in pit lakes that remain terminal sinks are predicted to increase linearly: for instance, an evaporation rate of 4 m/year sees the pit lake concentration increase to about 15 g/L within 100 years and about 70 g/L or 45 times Co within 500 years. In contrast, in throughflow systems, lake salinities will increase initially but will stabilize with time as the mine reaches a new, post‐mining hydraulic equilibrium. Scenarios with lower outflow rates plateau more slowly. For instance, concentrations increase to 15 and 45 g/L within 500 years with evaporation rates of 2 and 3 m/year, respectively, without reaching steady state. It is noteworthy that the stabilization of the concentrations happens long after the groundwater in‐ and outflow into the pit stabilizes (Figure S1). This is an important finding, as it highlights that a hydraulically stabilized post‐mining environment can still evolve geochemically over much longer timescales.

In model scenarios where density effects are considered, pit lake salinities reach a plateau after about 100 years. For net evaporation rates of 1, 2, 3, and 4 m/year, this occurs at salinities of about 4, 8, 12, and 15 g/L, respectively. These lake salinities are generally lower than under simulations that do not include density‐dependent flow, due to enhanced density‐driven lake outflow.

To reach a plateau of salinity in scenarios where throughflow occurs, all pit lake water needs to be exchanged while fluxes are already constant. The residence times of all pit lake water and with that the amount of evapoconcentration are then equal and the resulting pit lake concentration is constant.

**Figure 5:**
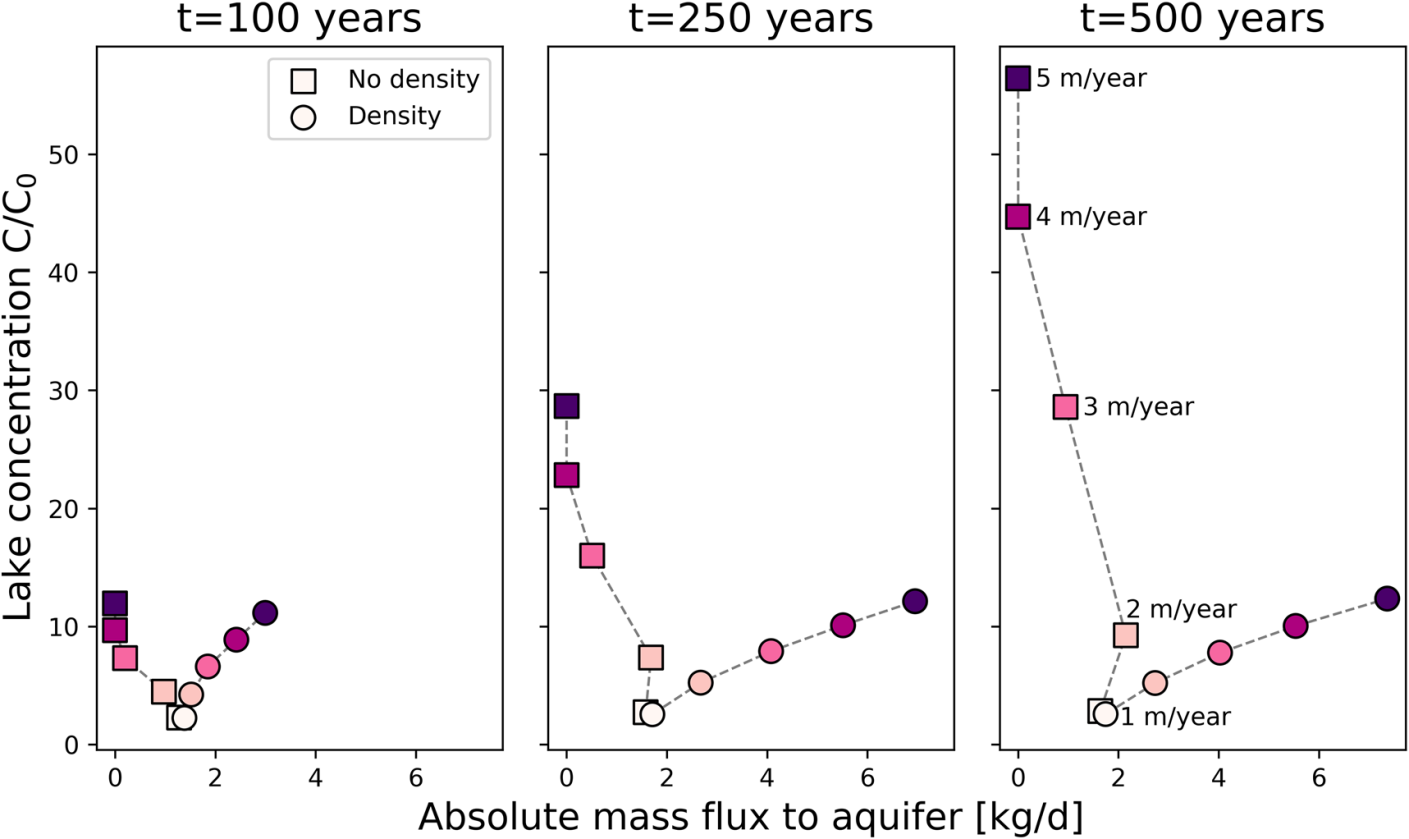
The concentration within the pit lake over the mass flux to the aquifer at three different time steps for different evaporation rates after mine closure.

Model simulations of lake salinity without consideration of density effects predict a negative correlation between lake concentrations and solute mass flux to the aquifer (i.e., high lake salinity corresponds to low mass flux to the aquifer; Figure 5). This trend, however, does not hold when density is considered. Concentrations within the lake increase less over time than in simulations that do not include density effects (Figure 4). However, the effect on the aquifer is significantly greater due to density‐driven outflow of mass from the pit lake. Thus, the greatest impact on the aquifer after 500 years is predicted for low evaporation rates (E = 1 and 2 m/year) when density effects are ignored with a solute mass flux of 1.6 and 2.2 kg/d from the lake to the aquifer, respectively. Here, the pit lake concentration stabilizes at about 4.4 g/L (E = 1 m/year) or 14 g/L (E = 2 m/year). Conversely, including the effects of density in model simulations results in greatest aquifer impacts under an evaporation rate of 5 m/year. This leads to a cumulative outflow of 1000 kt salt after 500 years and an absolute flux of 6.8 kg/d from pit lake to aquifer, while the lake concentration stabilizes at about 15 g/L. It is worth underscoring that the total amount of outflowing pit lake water (independent of concentration) is lowest when evaporation is highest (Figure S2) in scenarios both with and without consideration of density. However, as the concentration of pit lake outflow to the aquifer is significantly higher in scenarios with high net evaporation rates, so too is the outflowing absolute mass flux to the aquifer (compare Figures 5 and S3).

### Effect of Variable Density Flow on Impacted Aquifer Region

**Figure 6:**
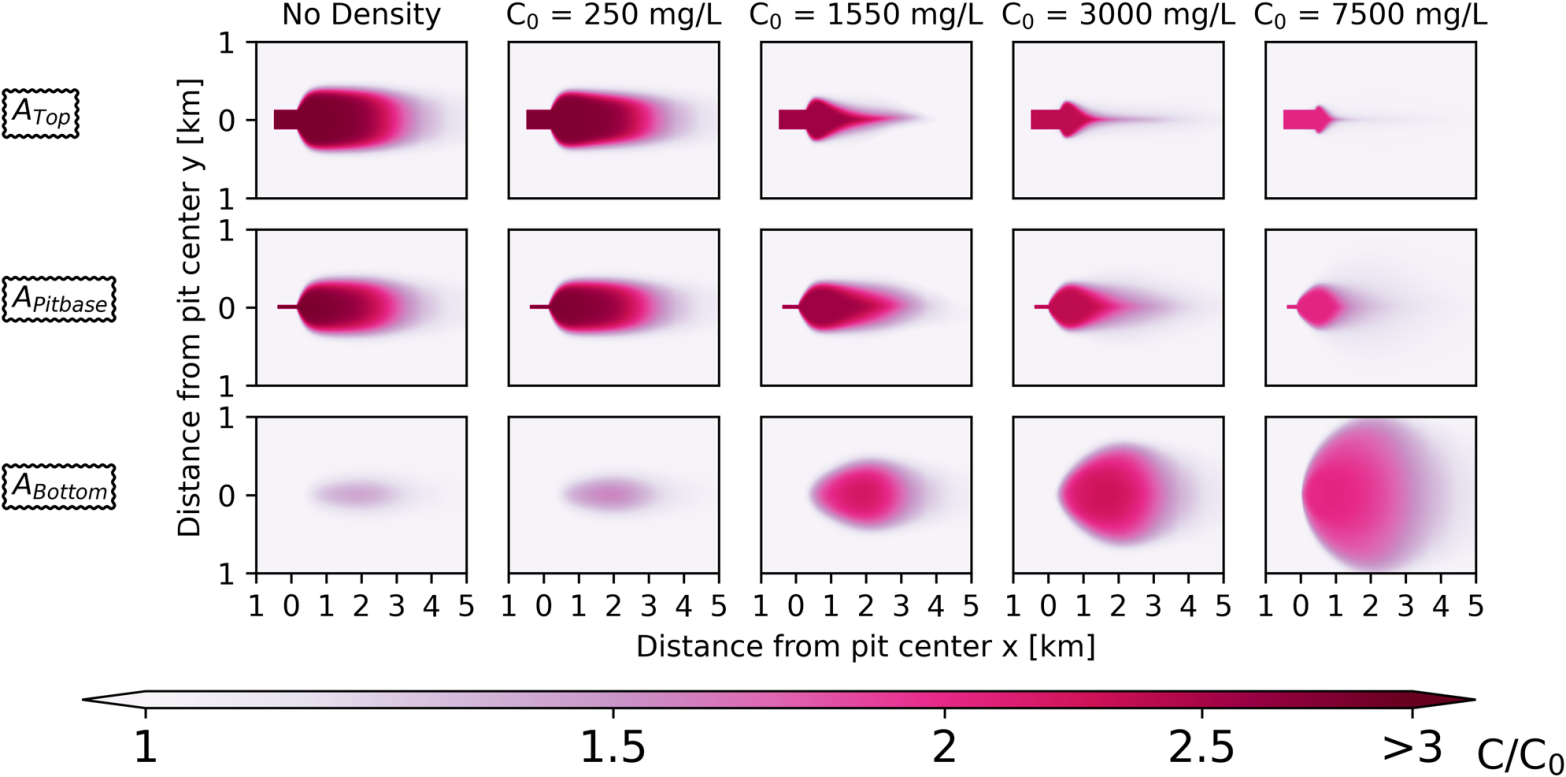
Plan view of salinity distribution patterns in the pit lake and the aquifer for scenarios with varying initial salinity concentrations (C_0_), and a scenario that was calculated without density‐dependent flow. Note that scenarios without density consideration have always the same relative concentrations independent of their initial concentration. The plume is plotted for *t* = 500 years after mine closure. The top of the aquifer (A_Top_) at the water table is about 25 m, the pit base (A_Pit base_) is at −65 m, and the bottom of the aquifer (A_Bottom_) is at about −200 m. The evaporation rate is 1 m/year for all scenarios.

Comparison of simulations shows that consideration of density in modeling of pit lakes has not only an impact on predicted pit lake and aquifer salinities. It is also impacting the region of aquifer affected. Outflow patterns, that is, the shape of the salinity plume, differ markedly between the scenarios, as shown in Figures 3 and 6. Where density is not considered or density effects are low, most water outflows relatively evenly over the entire pit wall. However, where density effects are significant, water outflow occurs more pronounced from the lower pit lake base and deeper parts of the aquifer are progressively more affected. Interestingly, density‐driven flow results in “candle flame”‐shape plumes in plan view within the shallow groundwater horizons (e.g., Figure 6 scenario with C_0_ >1550 mg/L; see Figure S4 for further scenarios). This “candle effect” is caused by denser water flowing downwards where flow velocities are not sufficiently high to overcome the buoyancy forces. Subsequently, deeper aquifer horizons are progressively more affected. In contrast, if density is not considered, or where ambient groundwaters are of low salinity, the deeper horizons of the aquifer are only marginally impacted.

### Impact of Various Hydrogeological Settings in Mine Environments

**Figure 7:**
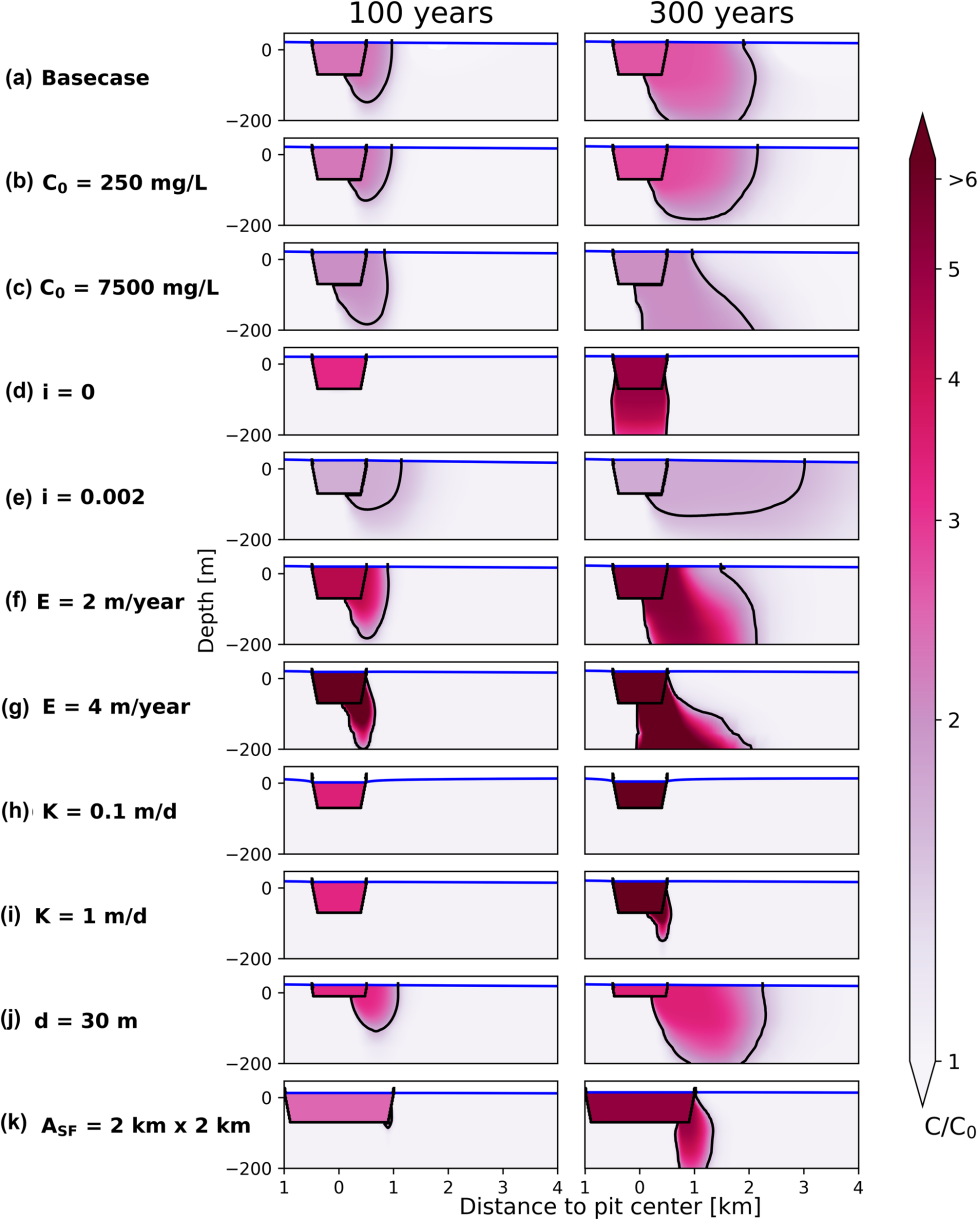
Cross‐section of salinity concentrations in various pit lake environments for 100 and 300 years after mining ceased. The base case (a) is set up as described in Table 1. All other scenarios see only one parameter changed, as indicated. All scenarios are calculated under the consideration of variable density flow. For easier comparison, isolines are shown for C = 1.6C_0_ (black line). C_0_ = initial concentration, i = regional hydraulic gradient, E = net evaporation, K = hydraulic conductivity, d = pit lake depth, A_SF_ = pit lake surface area.

Figure 7 shows the base case as well as multiple scenarios where one parameter was altered from the base case to determine their effects in isolation. Thereby, all scenarios are simulated with the consideration of variable density.

### Ambient Groundwater Salinities

Model simulations show that relatively fresh ambient groundwaters (e.g., 250 mg/L in Figure 7b) generally lead to a flow and salinity evolution that is virtually unchanged through density effects 500 years into the future. However, higher ambient groundwater concentrations lead to higher density differences between lake water and background groundwater concentration (Figure 7c). This leads to density‐driven enhanced outflow from the pit lake to the receiving groundwater (Figure S5). As this throughflow increases, the residence time within the pit decreases, leading to a decrease in pit lake salinity.

### Hydraulic Gradient

If there is no hydraulic background gradient and no consideration of density‐dependent flow, a pit lake will always remain a terminal sink. However, when density is considered, the influence of increasing salinity can provoke the pit to turn into a throughflow system. Accordingly, a model scenario without an ambient hydraulic gradient (pre‐mining) remains a terminal sink indefinitely under constant density assumptions (not shown). Under the consideration of varying density, this same pit lake remains a terminal sink for a prolonged time but eventually transitions into a throughflow system (Figures 7d, 7e, and S6). In this case, water leaves the pit only vertically under the evolving density gradient. This is of particular interest, as the assumption of constant density or pure flow simulations could easily lead to the erroneous assumption that the pit lake acts as a terminal sink for adverse pit lake water quality. In general, the higher the ambient hydraulic gradient, however, the lower the maximum concentration and the wider the spread into and closer to the surface of the aquifer.

### Evaporation

Model simulations demonstrate the strong effect of net evaporation on the evolution of salinity in and around pit lakes. Particularly for high evaporation rates, scenarios without density consideration see the pit lake concentration increase to a great extent, while scenarios where density is considered, generally impact the aquifer more. For the latter, outflow occurs from the bottom of the lake, while in simulations without density consideration the outflow occurs evenly along the whole pit wall (compare Figures 7f, 7g, S3, and S4). Interestingly, scenarios with high net evaporation rates that remained terminal sinks when density was not included may turn into throughflow systems when density is included. Between the scenarios with and without considering changes in density, significant differences in the pit lake concentration are noticeable.

### Hydraulic Conductivity

The hydraulic conductivity of the surrounding aquifer has a substantial impact on the dynamics of pit lake salinity. While in this study homogeneous aquifers are investigated, heterogeneity could alter plume behavior further.

Generally, the higher the hydraulic conductivity, the lower the maximum pit lake concentration and the wider the spread into the aquifer (Figures 7h, 7i, and S7). Scenarios with relatively low K_h_ values of 0.1 m/d remain terminal sinks for the entire simulation time of 500 years. Pit lake stages remain comparably low, and the water quality deteriorates significantly. Where the hydraulic conductivity is 1 m/d, pit lakes remain terminal sinks for 100 years but eventually transform into throughflow systems. While the amount of outflowing water is less for the case K = 1 m/d compared to the base case of K = 4 m/d, the water quality becomes worse. It is noteworthy that the vertical spread of salinity plumes significantly dependents on the anisotropy factor (Figure S7).

### Pit Lake Size

Testing model scenarios with varying pit depths and sizes identified key trends in salinity evolution and outflow behavior. With a decrease in pit depth the salinity concentration within the pit increases, as the evaporation surface makes up a larger proportion of the entire lake volume. Deeper pits lead to greater outflow and greater salt mass transfer into the aquifer driven by their larger pit volume and increased pit wall cross‐sectional area (Figures 7j, S8, and S9).

Larger pits (surface area 2 km × 2 km), lead to higher pit lake salinities, compared to the base case, as the longer residence time before the onset of outflow allowed greater evaporative concentration. When density effects are included, outflowing mass increases quickly and a strong impact on the surrounding aquifer can be observed (Figures 7k and S9).

In simulations where the pit was aligned orthogonally to the regional groundwater flow direction, salinity increases rise more sharply than in the base case; while outflowing pit lake water remains closer to shallower aquifer layers. This pattern is due to reduced upward flux through the pit base compared to the base case scenario. The base case pit lake functions as a conduit for the surrounding groundwater, resulting in deeper groundwater convergence towards the pit base, this effect is reduced for the orthogonal pit, due to the different pit base lengths in flow direction.

## Discussion

### Evaluating the Importance of Density Considerations

To establish whether density effects should be considered in numerical model predictions of post‐mining pit lake environments, it would be highly beneficial to know a‐priori whether a considered mine environment warrants the inclusion of variable density consideration or whether conservative transport predictions would suffice. The onset of instability due to density effects in groundwater systems has been investigated (e.g., under evaporation ponds) and analytical solutions have been developed to determine when and in which environmental settings this occurs (Wooding number: Wooding 1960; Wooding et al. 1997; or Rayleigh number: Rayleigh 1916; Simmons et al. 2001). The Wooding number, for instance, is a one‐dimensional ratio between convective and density gradients. It can be used to determine whether density gradients may suffice to induce outflow into the groundwater below an evaporation pond, and considers aquifer permeability, the density contrast between lake water and groundwater, and the upward flux velocity from the aquifer to the pond, relative to a critical Rayleigh number. It shows that if aquifer permeability or density gradients are sufficiently small, or if the hydraulic upward flux into the lake is sufficiently high, instability due to density may not occur. In our study, this would translate to ongoing terminal sink conditions for pit lakes. A comparison between three of our investigated pit lake scenarios to the Wooding number is shown in the supporting information (Appendix A, Figure S10). An overall satisfactory fit between our numerical simulations and the analytical framework can be observed.

It should be noted that analytical solutions such as the Wooding number calculations greatly enhance system understanding. However, since upward flow velocities into a pond or lake are difficult to measure in the field and cannot be inferred from conservative, density‐independent models, the practical applicability of this analytical framework is limited.

Evolving pit lakes are inherently transient systems, where water bodies deepen over time while the salinity increases. Under this transience, it is not possible to determine analytically when density‐induced pit lake outflow occurs and accordingly under which environmental settings density consideration becomes necessary to accurately predict a pit lake's hydraulic and water quality evolution. However, generic simulations such as those presented in this paper can help to provide guidance under which environmental conditions density effects are likely to be significant.

Sensitivity analysis suggests that the impact of density increases with increasing pit lake evaporation rate and ambient groundwater salinity, and with decreasing hydraulic conductivity and hydraulic gradient. Pit lakes that have high inflow rates relative to evaporation and low ambient groundwater concentrations are expected to be less impacted by density‐dependent flow.

**Figure 8:**
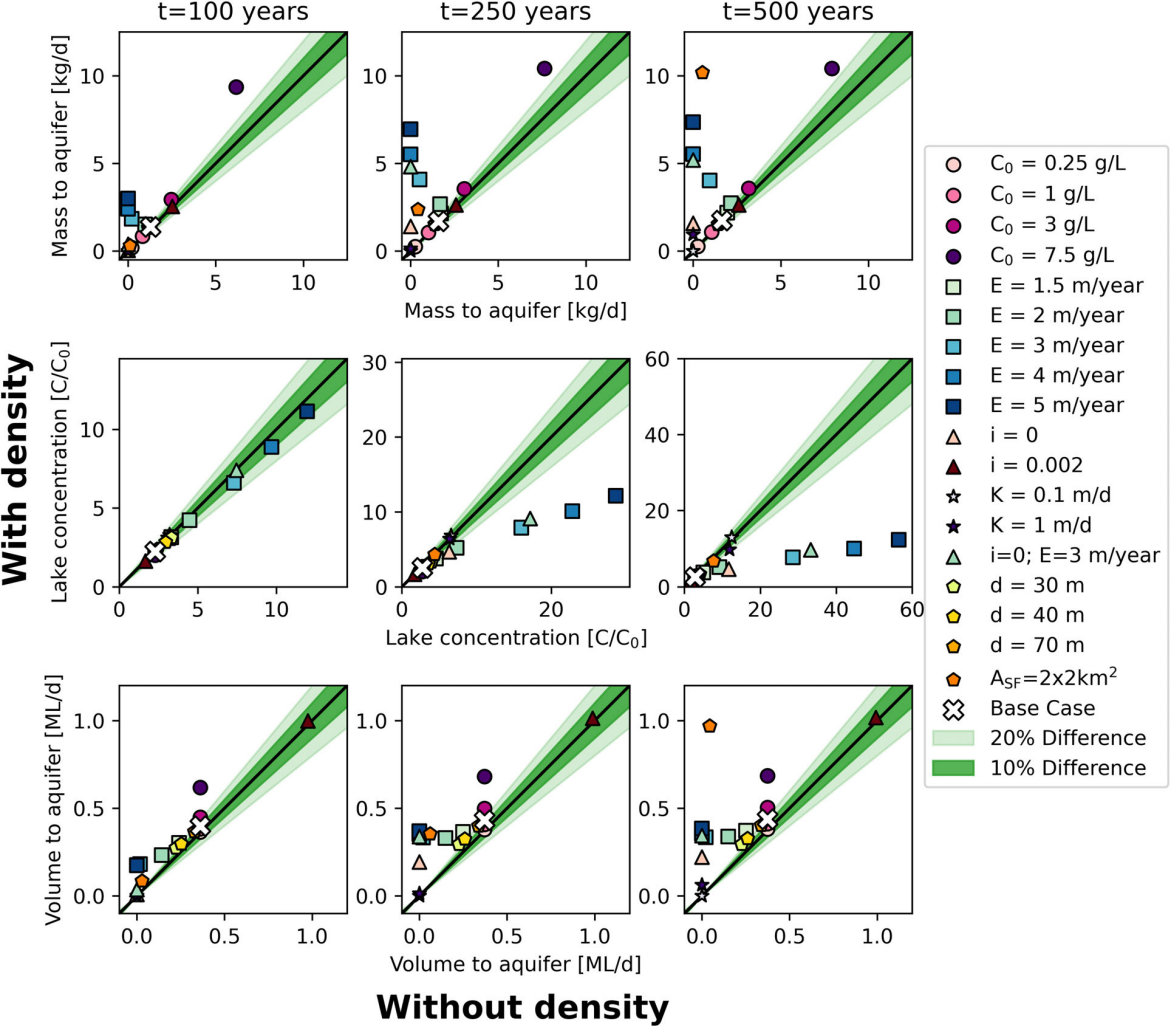
Comparison of simulations with density (*y*‐axis) and without density (*x*‐axis) in terms of outflowing mass to the aquifer, the relative lake concentration, and the volume to the aquifer for three timesteps.

The simulated model scenarios are compared in terms of outflowing mass to the aquifer, the relative lake concentration, and the volumetric pit lake water outflow into the aquifer (Figure 8). Simulation with C_0_ = 1550 mg/L, E = 1 m/year, K = 4 m/d and i = 0.001 produced less than a 20% difference between simulations with and without density for the pit lake salinity, the water flux from the pit lake to the aquifer and the solute mass flux from the pit lake to the aquifer within the first 250 years after mine closure. We would therefore expect that simulations with higher K or i or lower C_0_ or E values would also produce less than a 20% difference in these measures of lake concentration and pit lake–groundwater flux. Given the other uncertainties inherent in groundwater modeling, the additional required effort to include density‐dependent flow and the ensuing prolonged computational run times may not be warranted for such cases. Scenarios with high evaporation rates (other parameters as in base case conditions) have a large impact on outflowing mass from the pit lake even within relatively short time frames (100 years).

It should be pointed out that the simulation of density‐dependent flow is sensitive to spatial model discretization (Frolkovič and De Schepper 2000). A finer grid would potentially result in an earlier outflow of pit lake water into the less dense water lying below, that is, the ‘onset of instability’ would occur earlier the finer the discretization (Post and Simmons 2022). Also, relatively coarse grids may prevent so‐called ‘fingering’ to appear, where chaotic patterns form when denser water ingresses into fresher water (Simmons and Narayan 1997; Frolkovič and De Schepper 2000). Additional, numerical dispersion, more pronounced in coarser grids, can cause a wider spread of the plume and “washing out” of the plume boundaries (Konikow 2010). To assess grid resolution effects, three cross‐section models with comparable settings to the 3D model used in this study were tested at different discretization (dx = 12.5 m dz. = 10 m; dx = 3.125 m dz. = 2 m; dx = 1.5625 m dz. = 0.5 m, see Figure S11). It was observed that finer discretization resulted in slightly earlier onset of instability, however, the overall outflow patterns and simulated lake and aquifer salinities remained consistent across all cases. As such, while exact outflow times cannot be determined precisely, the here presented 3D model simulations provide an accurate depiction of the general flow dynamics that are altered by density consideration compared to the conservative transport model.

### Lake Stratification

In this study, the distribution of the salinity concentration within the emerging pit lake was uniform (perfect mixing). This is a valid approach for pit lakes that turn over at least once a year (holomictic conditions). Holomictic lakes may experience seasonal stratification; however, these are short‐term processes, that alone are not likely to have a major impact on the long‐term salinity evolution. Seasonal stratification occurs when warmer surface temperatures decrease water density in the upper lake layers, promoting stratification. Colder conditions and wind forces can overcome this density gradient, causing mixing (Boehrer and Schultze 2008). In arid to semiarid regions, high evaporation rates often lead to high net evaporation from lake surfaces (evaporation > [precipitation + runoff]), reducing the likelihood of permanent stratification (Jewell 2009). Evaporation from the upper layer of already salty lakes can additionally promote convective mixing that disrupts stratification (Bouffard and Wüest 2019). Thus, in arid and semiarid climates, pit lakes typically mix at least once a year, justifying the model assumption of perfect mixing. Examples of holomictic pit lakes in these climates are numerous and include Lake Kepwari, Western Australia (Salmon et al. 2017), former coal mines in the Hunter Basin, New South Wales, Australia and Bowen Basin, Queensland, Australia (Blanchette and Lund 2021), Enterprise Pit Lake, Northern Territory, Australia (Boland and Padovan 2002), gravel pit lakes at the Adriatic Coast, Ravenna, Italy (Mollema et al. 2015, note this is located in a Mediterranean not [semi]arid climate), and Lake Dexter, Nevada, USA (Castendyk et al. 2015).

In contrast, meromictic lakes, which are permanently stratified, constitute approximately 0.1% of lakes worldwide (Stewart et al. 2009). Pit lakes may be more susceptible to meromixis due to their often greater depth and wind sheltering due to high side walls, compared to natural lakes (Castendyk et al. 2015); however, geometric factors alone do not predict stratification (Castendyk and Webster‐Brown 2007; Schulze and Boehrer 2009). Meromictic conditions are more likely to occur in pit lakes receiving high surface runoff or having a permanent surface water connection. However, this study focusses on primarily groundwater‐fed pit lakes, which are known to be common around the world in arid to semiarid climates with net positive evaporation (Newman 2020; Johnson and Wright 2003; Kemanga et al. 2024). The formation of a stable freshwater layer atop denser saline water, that is, meromictic conditions, is unlikely in these environments. Therefore, assuming fully mixed pit lake salinities is reasonable for the climatic and hydrogeological scenarios considered in this study.

### Limitations

This study investigated the density effects on pit lake hydraulics to provide generalizable insights, as case studies are scarce due to the lack of field data availability. In order to achieve this aim the model set‐up was intentionally simplified, meaning aquifers had a constant background salinity, hydraulic conductivities were homogenous and pit surface areas were rectangular. Additionally, potential impacts from the buildup of a less permeable lakebed due to the precipitation of solids could decrease lake–groundwater interaction (e.g., Castendyk et al. 2015; Roemer et al. 2015); however, this was not considered in this study.

Arid or semiarid climates with a positive net evaporation rate, are considered in this chapter. The investigated unstable density conditions, provoked solely by evapoconcentration, would not occur for zero or negative net evaporation rates or if a connection to a surface water network is present. Indeed, under the given setup, negative net evaporation would always see pit lakes transition into throughflow systems, even without consideration of density.

As discussed earlier, perfect mixing within the pit lake is assumed. Thus, the lake's concentration would represent the mean salinity of the whole water body, representing the long‐term evolution of holomictic lakes. If permanent salinity stratification were to occur, the density close to the pit base would most likely increase, potentially enhancing density‐dependent pit lake water outflow from the pit base. To investigate meromictic lakes further or to account for seasonal stratification, it would be useful to couple the groundwater model with a lake model, facilitating the simulation of geochemical processes within the lake.

## Conclusions and Recommendations

This study identified mining environments with high groundwater background salinity, high evaporation rates or low to no hydraulic gradient generally require consideration of density‐driven flow to accurately predict salinity evolution within pit lake environments. Simulations that result in terminal sinks when density is not considered, such as those of low conductive geological formations, low background hydraulic gradients or those when evaporation is high, can in fact evolve into through‐flow systems when density‐driven flow is considered.

When density effects are considered, the additional outflow pressure of the denser lake water lying above the lighter groundwater generally leads to relatively lower pit lake salinities, but greater impacts on receiving groundwater systems.

For mine closure planning, it is important to investigate and evaluate potential density impacts. If density differences are likely to cause considerable changes in the pit lake salinity and/or pit lake water outflow into the aquifer, it is recommended to implement density‐dependent flow when modeling mine site closure. This could prevent unnoticed or underestimated contamination of the aquifer and thereby reduce environmental impacts when closing open pit mines.

A major barrier to improving predictions and developing better closure guidelines is the ongoing lack of publicly available, long‐term monitoring data from pit lakes. Monitoring programs should include continuous lake level and salinity measurements, capturing both seasonal variability and vertical stratification, if present. Monitoring bores should be installed to sufficient depth to capture the water fluxes that, despite existing close to the pit lake's surface, could sink to a considerable depth along the density gradients. If monitoring bores are too shallow, they might underestimate the amount of highly saline water discharging from the pit into the surrounding environment. In both closure planning and regulatory frameworks, the potential for density‐driven flow should not be overlooked. Properly accounting for it, through monitoring and modeling, can prevent unexpected long‐term impacts and support more sustainable and responsible mine closure outcomes.

## Supporting information


**Figure S1.** The groundwater inflow into the pit (black) and the pit lake concentration (orange) over 100 years post‐mine closure. For the simulation with (−‐) and without (−) density. While the groundwater flux plateaus within about 40 years, the salinity concentration within the pit lake requires significantly more time to stabilize (compare Figure 3 in main manuscript). Note that the sudden “steps” in the groundwater inflow are caused by the simulated lake stage reaching a new mining bench (a new layer of the model), leading to an abrupt change in the evaporative loss from the enlarged pit lake.
**Figure S2.** Cumulative in‐ and outflows [m^3^] from and to the pit for varying evaporation rates 500 years post mine closure.
**Figure S3.** Cross‐sectional view of salinity distribution patterns in the pit lake and the aquifer 100 years and 500 years post mining for different evaporation rates with and without considering density dependent flow in the numerical simulations. Initial concentration C_0_ = 1550 mg/L.
**Figure S4.** Plan‐view of salinity distribution patterns in the pit lake and the aquifer 500 years post mining for different evaporation rates with and without considering density dependent flow in the numerical simulations. Initial concentration C_0_ = 1550 mg/L. The top of the aquifer (A_Top_) at water table is about 25 m and the bottom of the aquifer (A_Bottom_) is at −200 m below surface.
**Figure S5.** The impact of density‐driven flow on salinity plumes and pit lake salinity for environments with varying initial groundwater concentrations (C_0_). All results are shown 500 years after mining ceased and relative to the initial concentration. Δρ marks the density difference between the pit lake water and the upstream, ambient groundwater.
**Figure S6.** Cross‐section view of salinity distribution patterns in the pit lake and the aquifer for different ambient hydraulic gradients 100 and 500 years after mine closure. Initial concentration C_0_ = 1550 mg/L. All scenarios include density‐driven flow.
**Figure S7.** Cross‐section of salinity distribution patterns in the pit lake and the aquifer for different hydraulic conductivities 100 and 500 years after mine closure. Initial concentration C_0_ = 1550 mg/L. All scenarios include density‐driven flow.
**Figure S8.** Cross‐section of salinity distribution patterns in the pit lake and the aquifer for different pit lake sizes 500 years after mine closure, for scenarios with and without density. Initial concentration C_0_ = 1550 mg/L. The surface area of the large pit is 2 km × 2 km.
**Figure S9.** Fluxes and pit lake concentration for pit with different geometries.
**Figure S10.** Comparison between numerical model simulations and the Wooding number. Each dot represents one output time of the numerical simulation. A close match between simulations and analytical predictions occurs when the first outflow point (blue dot) aligns with the critical threshold (black line). The scenarios presented are: (a) Kx = 4 m/d, Kz = 0.4 m/d, c0 = 1550 mg/L, net E = 1 m/year, No background gradient; (b) Kx = 4 m/d, Kz = 0.4 m/d, c0 = 1550 mg/L, net E = 3 m/year, No background gradient; (c) Kx = 0.1 m/d, Kz = 0.01 m/d, c0 = 3000 mg/L, net E = 1 m/year, background gradient = 0.001. Further description in Appendix A1.
**Figure S11.** Onset of instability [left] and cumulative mass to aquifer [right] for 2D cross sections with varied discretization. Net evaporation was set to 1 m/year and C_0_ = 5000 mg/L. Pit depth is 30 m.
**Appendix A.** Comparison to Wooding number.

## Data Availability

The data that support the findings of this study are available from the corresponding author upon reasonable request.
