# Supplementary material for: When Density Matters: Hydraulic and Salinity Evolution in Groundwater‐Fed Pit Lakes in Semiarid and Arid Climates
Source: Ground Water. 2026 May 7;64(4):441–56. doi: 10.1111/gwat.70074 (PMC13353559; doi:10.1111/gwat.70074)
Supplement: Supplementary file 1 — Figure S1. The groundwater inflow into the pit (black) and the pit lake concentration (orange) over 100 years post‐mine closure. For the simulation with (−‐) and without (−) density. While the groundwater flux plateaus within about 40 years, the salinity concentration within the pit lake requires significantly more time to stabilize (compare Figure 3 in main manuscript). Note that the sudden “steps” in the groundwater inflow are caused by the simulated lake stage reaching a new mining bench (a new layer of the model), leading to an abrupt change in the evaporative loss from the enlarged pit lake. Figure S2. Cumulative in‐ and outflows [m3] from and to the pit for varying evaporation rates 500 years post mine closure. Figure S3. Cross‐sectional view of salinity distribution patterns in the pit lake and the aquifer 100 years and 500 years post mining for different evaporation rates with and without considering density dependent flow in the numerical simulations. Initial concentration C0 = 1550 mg/L. Figure S4. Plan‐view of salinity distribution patterns in the pit lake and the aquifer 500 years post mining for different evaporation rates with and without considering density dependent flow in the numerical simulations. Initial concentration C0 = 1550 mg/L. The top of the aquifer (ATop) at water table is about 25 m and the bottom of the aquifer (ABottom) is at −200 m below surface. Figure S5. The impact of density‐driven flow on salinity plumes and pit lake salinity for environments with varying initial groundwater concentrations (C0). All results are shown 500 years after mining ceased and relative to the initial concentration. Δρ marks the density difference between the pit lake water and the upstream, ambient groundwater. Figure S6. Cross‐section view of salinity distribution patterns in the pit lake and the aquifer for different ambient hydraulic gradients 100 and 500 years after mine closure. Initial concentration C0 = 1550 mg/L. All scenarios include density‐drive [file GWAT-64-441-s001.docx]

Supporting Information for

When density matters: hydraulic and salinity evolution in groundwater-fed pit lakes in semi-arid and arid climates

Birte Moser^1^, Peter Cook^1^, Janek Greskowiak^2^, Ilka Wallis^1^

^1^ Flinders University, College of Science and Engineering, National Centre for Groundwater Research and Training, Adelaide, South Australia, P.O. Box 2100, Adelaide, South Australia 5001, Australia

^2^ Department of Biology and Environmental Sciences, Carl-von-Ossietzky University of Oldenburg, Ammerländer Heerstraße 11, Oldenburg D-26129, Germany

**Contents of this file**

Figures S1 to S11, Appendix A1

**Introduction**

The supplementary figures provide additional comparisons of model simulations and volumetric fluxes from and to the emerging pit lake. As in the main manuscript described, models were set-up and results were post-processed using FloPy version 3.4.2 (Bakker et al., 2024) and Matplotlib version 3.5.2 (Hunter, 2007).


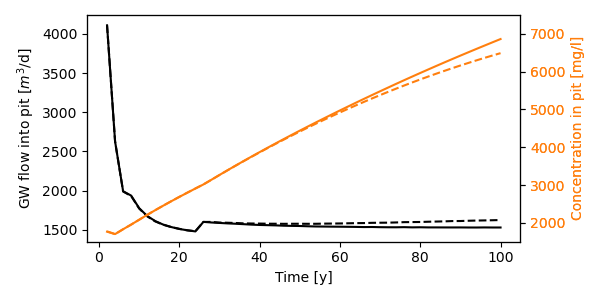


Figure S1. The groundwater inflow into the pit (black) and the pit lake concentration (orange) over 100 years post-mine closure. For the simulation with (--) and without (-) density. While the groundwater flux plateaus within about 40 years, the salinity concentration within the pit lake requires significantly more time to stabilise (compare Figure 3 in main manuscript). Note that the sudden “steps” in the groundwater inflow are caused by the simulated lake stage reaching a new mining bench (a new layer of the model), leading to an abrupt change in the evaporative loss from the enlarged pit lake.


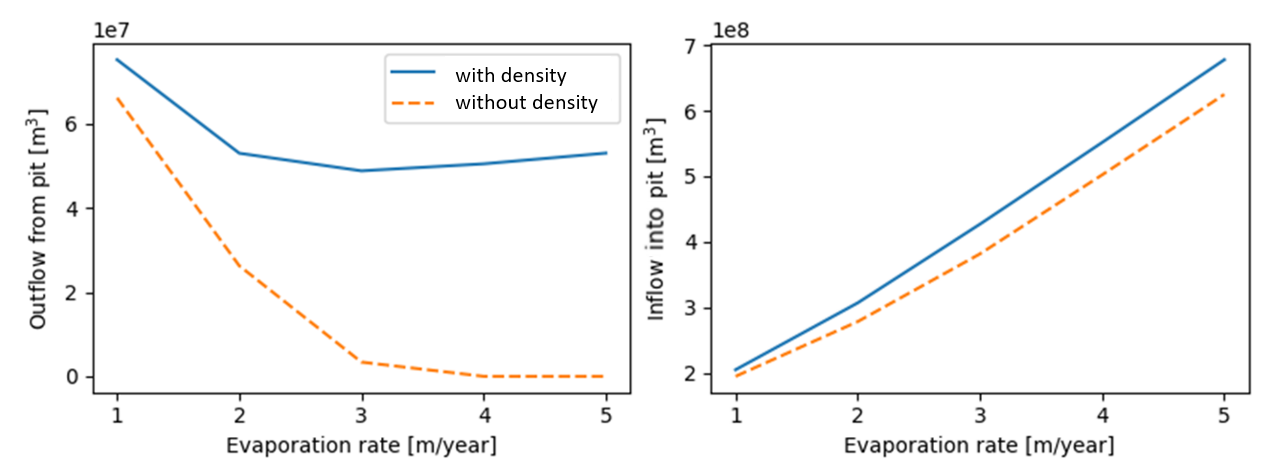


**Figure S2**: Cumulative in- and outflows [m^3^] from and to the pit for varying evaporation rates 500 years post mine closure.

**
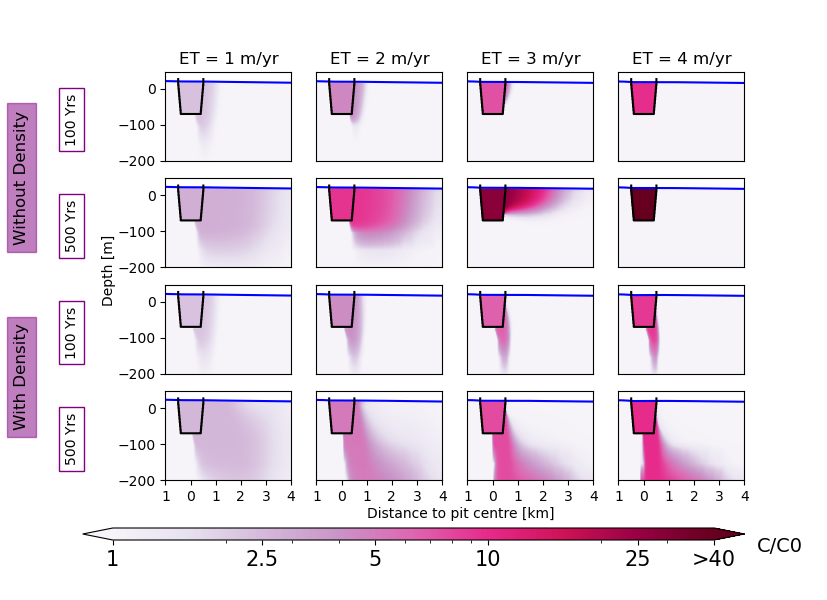
**

**Figure S3:** Cross-sectional view of salinity distribution patterns in the pit lake and the aquifer 100 years and 500 years post mining for different evaporation rates with and without considering density dependent flow in the numerical simulations. Initial concentration C_0_=1550 mg/L.


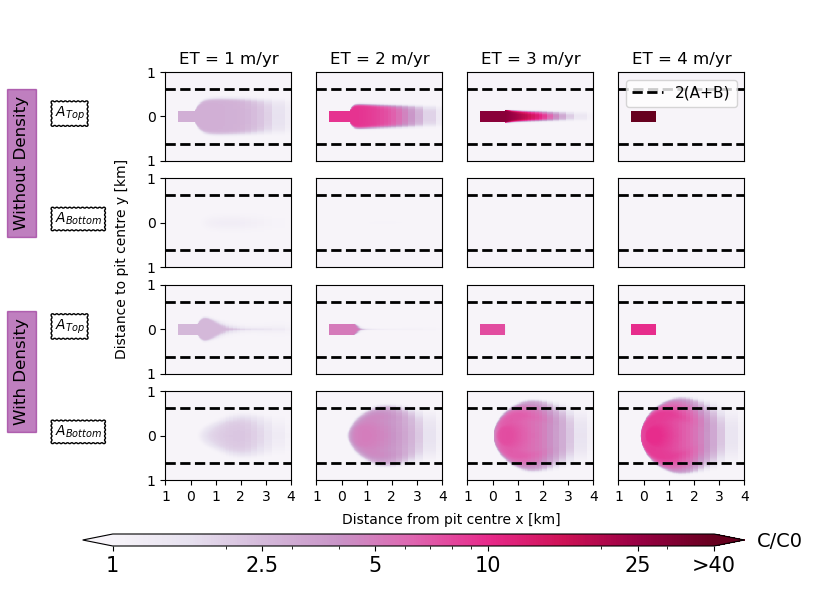


**Figure S4:** Plan-view of salinity distribution patterns in the pit lake and the aquifer 500 years post mining for different evaporation rates with and without considering density dependent flow in the numerical simulations. Initial concentration C_0_=1550 mg/L. The top of the aquifer (A_Top_) at water table is about 25 m and the bottom of the aquifer (A_Bottom_) is at -200m below surface.


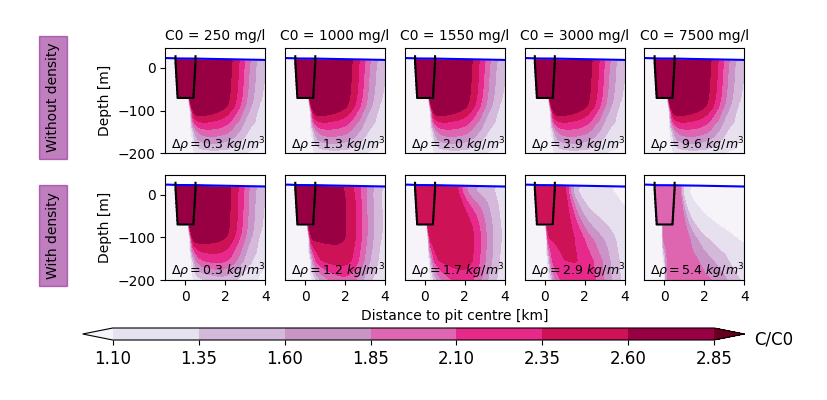


**Figure S5:** The impact of density-driven flow on salinity plumes and pit lake salinity for environments with varying initial groundwater concentrations (C_0_). All results are shown 500 years after mining ceased and relative to the initial concentration. Δρ marks the density difference between the pit lake water and the upstream, ambient groundwater.


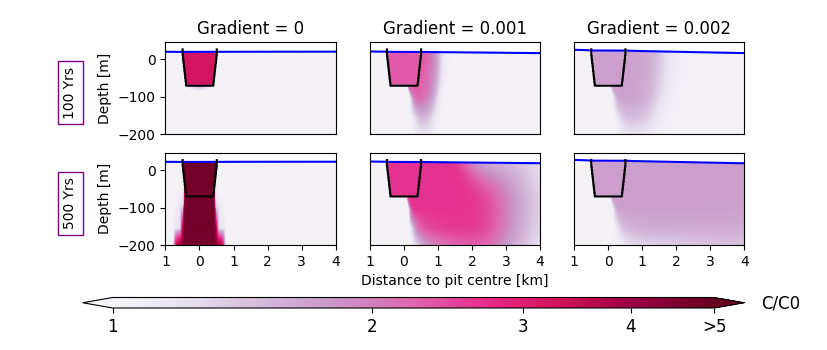
 **Figure S6:** Cross-section view of salinity distribution patterns in the pit lake and the aquifer for different ambient hydraulic gradients 100 and 500 years after mine closure. Initial concentration C_0_=1550 mg/L. All scenarios include density-driven flow.


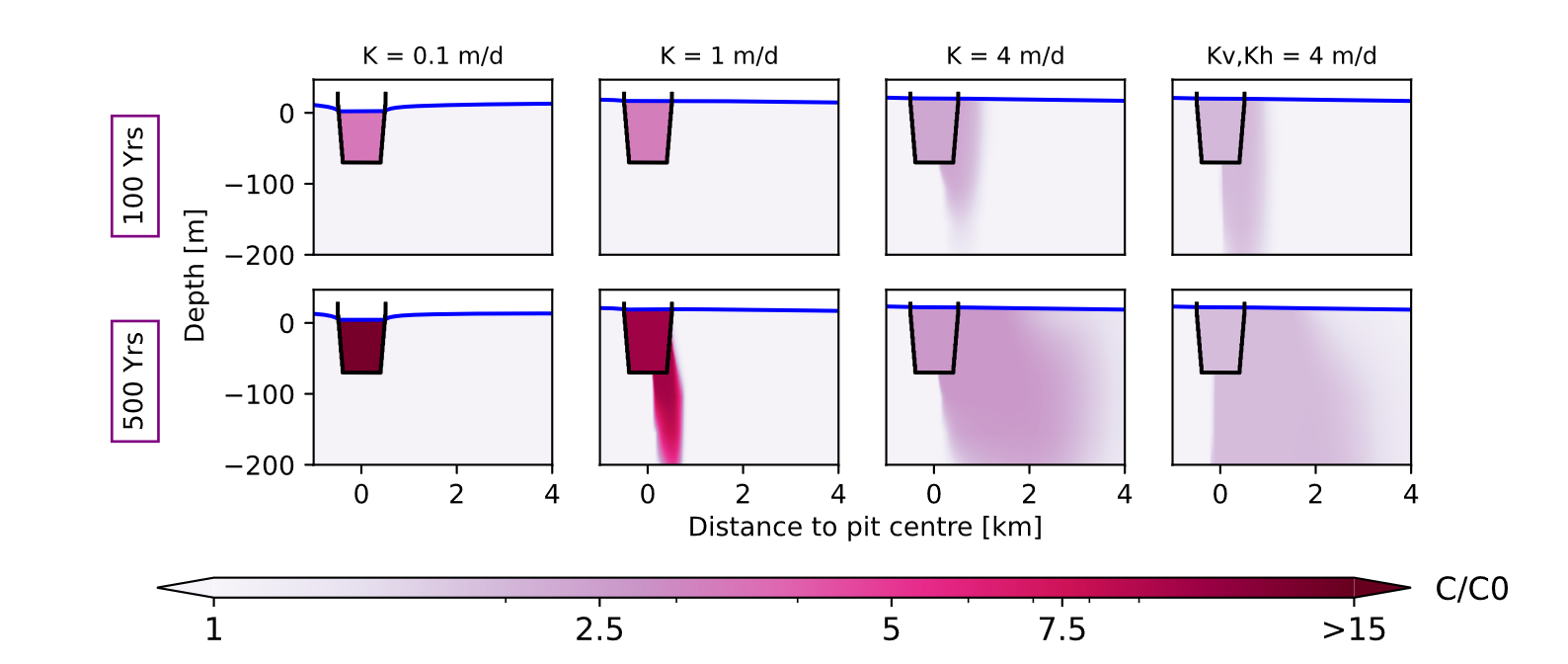


**Figure S7:** Cross-section of salinity distribution patterns in the pit lake and the aquifer for different hydraulic conductivities 100 and 500 years after mine closure. Initial concentration C_0_=1550 mg/L. All scenarios include density-driven flow.


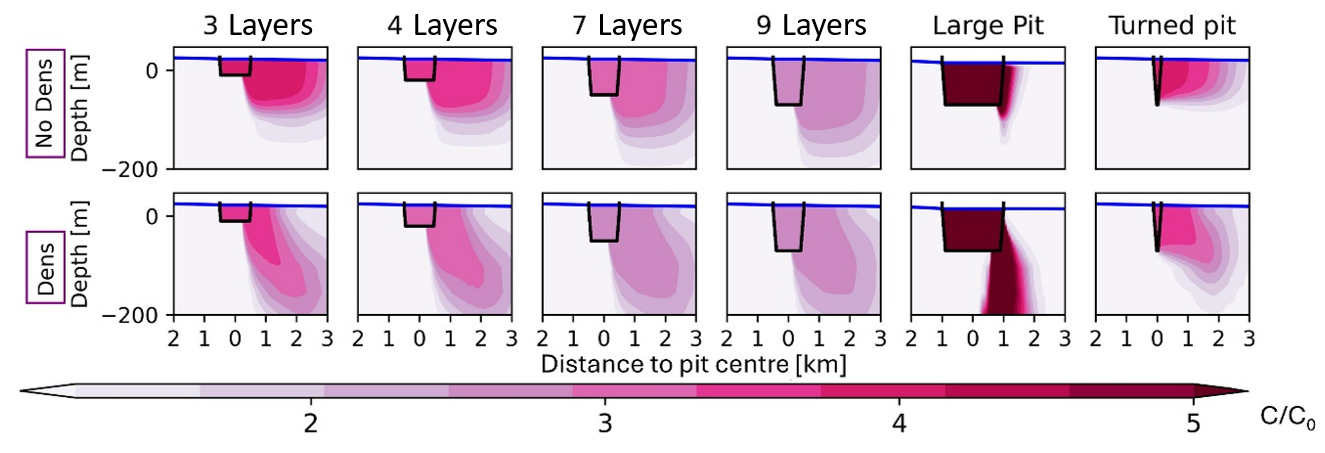


**Figure S8:** Cross-section of salinity distribution patterns in the pit lake and the aquifer for different pit lake sizes 500 years after mine closure, for scenarios with and without density. Initial concentration C_0_=1550 mg/L. The surface area of the large pit is 2 km x 2 km.

***
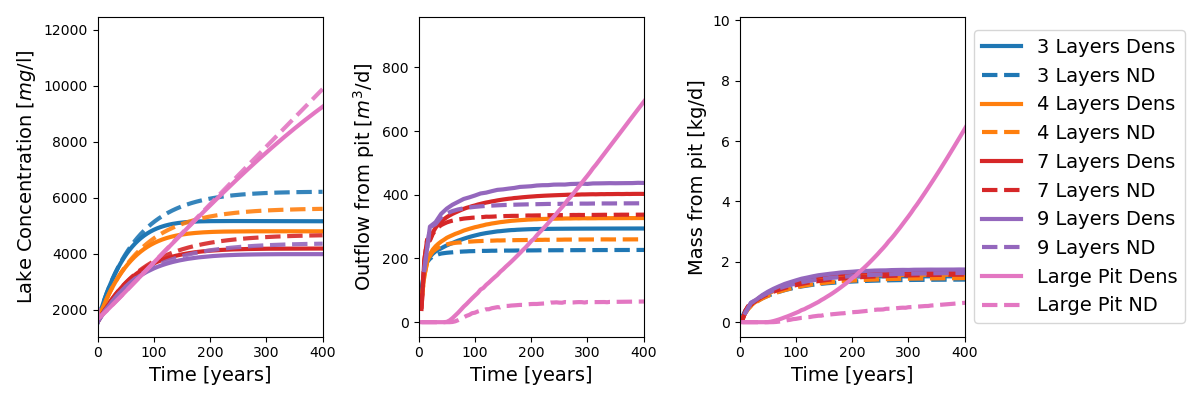
***

**Figure S9:** Fluxes and pit lake concentration for pit with different geometries.


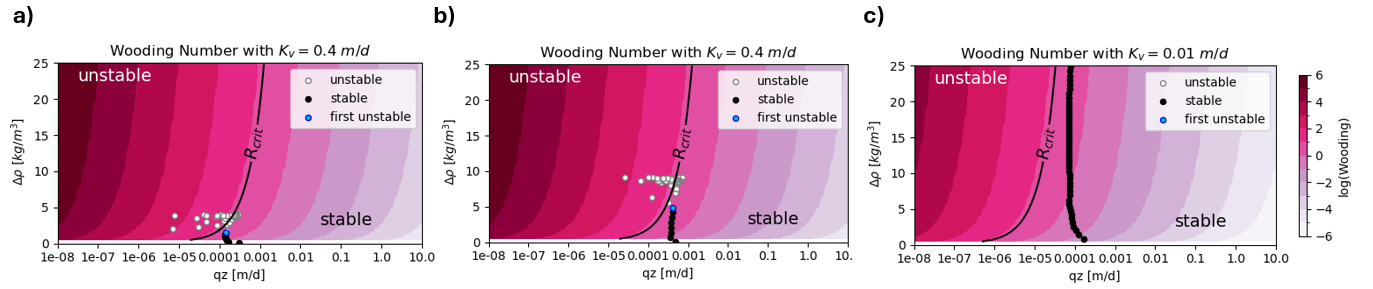


**Figure S10:** Comparison between numerical model simulations and the Wooding number. Each dot represents one output time of the numerical simulation. A close match between simulations and analytical predictions occurs when the first outflow point (blue dot) aligns with the critical threshold (black line). The scenarios presented are: a) Kx=4 m/d, Kz = 0.4 m/d, c0 = 1550 mg/L, net E = 1 m/year, No background gradient; b) Kx=4 m/d, Kz = 0.4 m/d, c0 = 1550 mg/L, net E = 3 m/year, No background gradient; c) Kx=0.1 m/d, Kz = 0.01 m/d, c0 = 3000 mg/L , net E = 1 m/year, background gradient =0.001. Further description in Appendix A1.

**
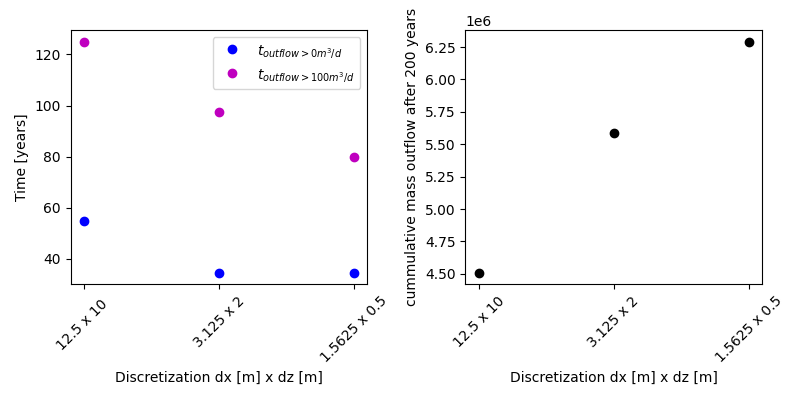
**

**Figure S11:** Onset of instability [left] and cumulative mass to aquifer [right] for 2D cross sections with varied discretization. Net evaporation was set to 1 m/year and C_0_ =5000 mg/L. Pit depth is 30 m.

**Supplementary Appendix A**: Comparison to Wooding number

Our findings under which conditions density dependent flow should be considered in a numerical model, and when it can be neglected, are broadly consistent with predictions based on the Wooding number. Wooding (1960) identified a critical Rayleigh number (𝑅𝑎^𝛿^_cr_ ≈7) that marks the onset of instability - in this context, the first occurrence of outflow from the evaporating lake (Post & Simmons, 2022). The Wooding number is expressed as:

$${Ra}^{\delta}=\frac{\Delta\rho gk}{{\mu q}_{z}}$$

where qz is vertical flow velocity, Δρ is the density contrast between the lake and the groundwater, g gravitational acceleration, k the aquifer’s intrinsic permeability, μ and the dynamic viscosity.

Larger Rayleigh numbers thereby indicate a greater tendency for density-driven instabilities to develop. These become more likely as upward flux decreases, as density contrasts increase, or as aquifer permeability increases.

While the Wooding number provides a useful criterion, it does not account for background hydraulic gradients, anisotropy, or transient effects. In contrast, the 3D numerical model developed in this study incorporates these processes. To approximate a comparison between the analytical Wooding number approach and the 3D numerical model simulations, we compared three scenarios:

a) K_x_=4 m/d, K_z_ = 0.4 m/d, c0 = 1550 mg/L, net E = 1 m/year, No background gradient, and

b) K_x_=4 m/d, K_z_ = 0.4 m/d, c0 = 1550 mg/L, net E = 3 m/year, No background gradient, and

c) K_x_=0.1 m/d, K_z_ = 0.01 m/d, c0 = 3000 mg/L, net E = 1 m/year, background gradient =0.001.

For consistency with the model setup, permeability was replaced with hydraulic conductivity, assumed constant despite variations in viscosity or density. The Wooding number was reformulated as:

$${Ra}^{\delta}=\frac{\Delta\rho K}{{\rho q}_{z}}.$$

qz represents the minimum upward specific discharge at the pit base, and Δρ the density contrast between pit lake and groundwater, K is the vertical hydraulic conductivity K_z_. Data points for qz and Δρ were plotted for each numerical model output time (Figure S10) and classified as stable (no pit lake outflow), first unstable (onset of outflow), or unstable (sustained outflow). A close match between simulations and analytical predictions occurs when the first outflow point aligns with the critical threshold (black line, 𝑅𝑎^𝛿^_cr_ ≈7).

Overall, the three tested scenarios show good agreement with the Wooding analytical framework. Over time, ongoing evapoconcentration increases density contrasts while flattening gradients reduce upward flux, together favoring pit lake to aquifer outflow (Figure S10a, b). However, in low-conductivity settings, the system may remain stable indefinitely because density contrasts never reach the threshold required for outflow (Figure S10c). Note that episodic reductions in net evaporation (e.g., from runoff events) could further reduce upward fluxes, potentially transforming even stable terminal sink lakes periodically into throughflow systems. It is worth emphasizing that the outcomes from the analytical Wooding number calculations will deviate from the numerical model outputs when background hydraulic gradients become important, given that the latter is not considered in the Wooding number calculations.

It is important to note that it was attempted to apply the Wooding number framework while using vertical flow velocities from conservative simulations (no density consideration). However, these did not satisfactorily reproduce the velocities obtained from simulations that did consider density. Consequently, the analytical framework based on the Wooding number could not be used to determine, a priori, whether density effects would influence system stability.
